# PROCOV: maximum likelihood estimation of protein phylogeny under covarion models and site-specific covarion pattern analysis

**DOI:** 10.1186/1471-2148-9-225

**Published:** 2009-09-08

**Authors:** Huai-Chun Wang, Edward Susko, Andrew J Roger

**Affiliations:** 1 Department of Mathematics and Statistics, Dalhousie University, Halifax, N.S. B3H 3J5, Canada; 2 Department of Biochemistry and Molecular Biology, Dalhousie University, Halifax, N.S. B3H 1X5, Canada; 3 Centre for Comparative Genomics and Evolutionary Bioinformatics, Dalhousie University, Canada

## Abstract

**Background:**

The covarion hypothesis of molecular evolution holds that selective pressures on a given amino acid or nucleotide site are dependent on the identity of other sites in the molecule that change throughout time, resulting in changes of evolutionary rates of sites along the branches of a phylogenetic tree. At the sequence level, covarion-like evolution at a site manifests as conservation of nucleotide or amino acid states among some homologs where the states are not conserved in other homologs (or groups of homologs). Covarion-like evolution has been shown to relate to changes in functions at sites in different clades, and, if ignored, can adversely affect the accuracy of phylogenetic inference.

**Results:**

PROCOV (protein covarion analysis) is a software tool that implements a number of previously proposed covarion models of protein evolution for phylogenetic inference in a maximum likelihood framework. Several algorithmic and implementation improvements in this tool over previous versions make computationally expensive tree searches with covarion models more efficient and analyses of large phylogenomic data sets tractable. PROCOV can be used to identify covarion sites by comparing the site likelihoods under the covarion process to the corresponding site likelihoods under a rates-across-sites (RAS) process. Those sites with the greatest log-likelihood difference between a 'covarion' and an RAS process were found to be of functional or structural significance in a dataset of bacterial and eukaryotic elongation factors.

**Conclusion:**

Covarion models implemented in PROCOV may be especially useful for phylogenetic estimation when ancient divergences between sequences have occurred and rates of evolution at sites are likely to have changed over the tree. It can also be used to study lineage-specific functional shifts in protein families that result in changes in the patterns of site variability among subtrees.

## Background

The covarion hypothesis of molecular evolution proposes that selective pressures on a given amino acid or nucleotide site are dependent on the identity of other sites in the molecule that change throughout time, resulting in changes of evolutionary rates of sites along the branches of a phylogenetic tree [1]. At the sequence level, covarion-like sites are often recognizable in alignment columns as residues that are conserved among taxa in one clade but variable among taxa in one or several other clades. Changes in rates at sites in different sequences are also referred to as type-I functional divergence [2] or 'heterotachy' [3]. Covarion-like evolution is now widely recognized as an important mode of molecular evolution in protein-coding genes, structural RNA, and DNA regulatory elements (*e.g*., [4-6]). This realization has fueled the development of several kinds of phylogenetic models including: (i) 'covarion models' that are designed to model the stochastic changes of rates at sites in sequences [7-12], (ii) discrete 'rate-shift' models where sudden changes in rates at multiple sites occur at particular splits in the tree [13], and (iii) mixture of branch lengths-based heterotachy models [14-18]. Empirical studies have shown that phylogenetic estimation under the covarion models may recover different optimal topologies than when estimation is performed ignoring covarion effects [*e.g*., [10]]. Indeed, simulation studies have shown that under some branch-length conditions, use of rates-across-sites (RAS) models that ignore covarion effects may cause long-branch repulsion biases in the resulting phylogenetic estimates [19]. Other studies have focused on developing computational methods to detect whether covarion-like evolution occurs in protein families [20-22], identify covarion or heterotachous sites to analyse functional shifts in a protein family [2,13,23-28] and detect positive selection in primate and viral genes [28-31].

Covarion models with changing rates of evolution at sites in different parts of the tree build upon the simpler RAS models that assume evolutionary rates are variable among sites but constant across lineages in a gene or protein. RAS is typically modeled by a 'discretized' approximation of the gamma distribution with a series of equiprobable rate classes [32]. The modeling of covarion processes is more challenging. Typically, these models allow rates at a site to vary gradually through the tree according to a stochastic process. The gradual rate shift in a covarion context can be formulated as a Markov model of rate switching between different rate classes, usually eight or less.

Five specific covarion models have been proposed that differ in the complexity of the rate switching processes [7-12]. The simplest model, proposed by Tuffley and Steel [7], assumes that rates along a branch in a phylogenetic tree can have two states 'off' and 'on'; switching from 'off' to 'on' occurs with one rate (s01) and from 'on' to 'off' (s10) with another rate. When a site is 'off', no substitutions occur and when it is 'on', substitutions occur at a constant rate. Huelsenbeck [8] added additional rate classes to this model. In the Huelsenbeck model, when the site is 'on', the expected substitution rate per unit time at the site is a specific rate drawn from the discrete gamma distribution, whereas it is zero when it is 'off'. A third covarion model was developed by Galtier [9], who assumed that only a subset of sites (of fixed proportion, B) evolve under the covarion process. The remaining sites have a site-specific rate drawn from a discrete gamma distribution. For sites evolving under the covarion process, rates are also drawn from a gamma distribution and the different rate classes can switch freely between each other at a single rate (s11). A more general model that combines features of both the Tuffley-Steel/Huelsenbeck models and the Galtier model was recently proposed [10], in which a covarion site may not only switch between an 'on' and 'off' state but also can switch between different rate categories of 'on' states. This latter model allows a variety of switching rates between the rate classes. More recently Whelan proposed a further generalized model which allows substitution rate-matrix changes as well as rate switches along the tree branches [[11], see also [12]].

The first four covarion models are described in Wang et al. (2007) [10] which were implemented in PROCOV for maximum likelihood (ML) estimation of covarion parameters for a fixed phylogenetic tree and protein alignment data. The new version of PROCOV described herein allows ML-based tree estimation using the subtree-pruning regrafting (SPR) algorithm, under a variety of amino acid substitution models including JTT, WAG and LG [33-35]. We have also utilized several numerical libraries in PROCOV to improve the efficiency of the likelihood calculations and thereby make computationally intensive tree searching analyses more practical. Here we demonstrate the utility of PROCOV in performing 'deep-level' phylogenomic analyses where model misspecification can often lead to long-branch attraction. We further explore the use of PROCOV as a way to detect covarion sites in protein families that have structural and functional significance.

## Implementation

As in all common likelihood-based methods, PROCOV implements a pruning algorithm [36] for the likelihood calculation. In conventional Markov models of protein evolution, there are 20 amino acid states and the substitution rates of the amino acids are described by an instantaneous substitution rate matrix (a *Q *matrix), such as the JTT model. Under the covarion model, character states are two dimensional, describing both the amino acid state and the substitution rate at that state at any given time. The *Q *matrix in a covarion model is thus a large sparse matrix. In PROCOV we used an algorithm introduced in [37] to decompose the *Q *matrix into a sum of two Kronecker products, each consisting of two smaller matrices. Even with this efficient algorithm, the calculation of the likelihood of the data for a given tree with the general covarion model is about 10 to 20 fold slower than for an RAS model with the same number of rate categories. This is because likelihood calculations under the general covarion model have a much larger number of terms to be summed over at each ancestral node as compared to an RAS model. For instance, under the general covarion model with 4 rates, there is a 16-fold increase in the number of terms to be summed relative to that under the RAS model.

For a given topology, ML estimates of parameters are obtained by a modified Newton-Raphson algorithm which requires the calculation of derivatives of the likelihood function with respect to each adjustable parameter. As analytical derivatives are difficult to compute for the covarion parameters, numerical derivatives are computed for all three covarion switching parameters. The derivative for the proportion of covarion sites parameter π in the general and Galtier models is computed analytically as the difference of the covarion likelihood and RAS likelihood across the sites. For the tree searching function, we used the SPR algorithm implemented in NHML [38]. An initial tree is modified by pruning subtrees and moving them to other places. If a rearrangement results in an increase of the likelihood, that tree is kept as a starting tree. The algorithm iterates until no rearrangement increases the likelihood.

PROCOV is written in ANSI C, and is based on the phylogenetic inference package NHML [9,38]. The current version of PROCOV needs a user-supplied starting tree which should be rooted; the "retree" program of PHYLIP [39] can be used for re-rooting. The starting tree can, for instance, be a neighbor-joining tree or a parsimony tree available from most phylogenetic packages. Compared with NHML, PROCOV has numerous new features, including, for instance, a command-line argument for setting models, parameters, input and output data; implementing protein models and four covarion models (NHML only implements the Galtier model for DNA data); new functions for matrix decomposition, matrix operations and computing derivatives. We have also introduced the following algorithms to speed up the tree searching procedure. Since the optimization of the gamma shape parameter (α) and the covarion parameters takes time, during the tree searching stage, we re-optimize these parameters only when a tree with a higher likelihood than the previous best tree is found. In this way, these parameters drift to the optimal values as the search proceeds. Furthermore, we relax the convergence condition to optimize parameters during the tree search stage; parameter optimization stops when the log-likelihood difference between two consecutive iterations is less than 0.1. For optimizing the final optimal tree, we impose a much stricter constraint (log-likelihood difference = 0.0001). Although the likelihood gain from a stricter convergence threshold is usually small (less than 1), according to our simulation results, this threshold yields parameter values much closer to their true values.

Some of the NHML routines are particularly useful for saving tree searching time and so have been inherited by PROCOV. For example, if a starting tree in the Newick format contains high bootstrap values that are greater than the maximum bootstrap value allowed for branch move during the SPR searches (defined by the variable SH_MAXBOOTCROSSED in the option files of the PROCOV source code package), those branches will not move separately in the SPR stage. Similarly it also has a function to forbid moving those branches that are longer than a user-defined value (defined by the variable SH_MAXLCROSSED in the option files). This branch movement restriction, resulting in partial SPR searches, gives user the flexibility in choosing which internal nodes are fixed and therefore can greatly reduce tree search time if many nodes are fixed. An extreme form of this branch movement restriction is to restrict PROCOV to compare only a few competing topologies, as in our previously published analyses of Angiosperm phylogeny (see [10]). Furthermore, PROCOV inherits from NHML a 'restart' function that can save all of the currently evaluated trees so that it will automatically bypass those topologies if the program has to be started over again. These functions are of practical importance as ML estimation under the general covarion model will usually take several days for a moderate-sized dataset (e.g., 30 taxa 400 sites).

For compilation of the source code, we recommend the use of GCC or compatible compilers. Use of the -O3 and -funroll-loops for compiler optimization also significantly increases its running speed; for a small dataset we tested, this speedup can be more than two fold. PROCOV spends a lot of time doing matrix operations, such as matrix multiplication, matrix inversion and eigenvalue/eigenvector decomposition. To do these kinds of calculations, phylogenetic programs including NHML commonly use C routines based on those described in *Numerical Recipes *[40]. To improve speed, the current version of PROCOV makes use of the high quality routines in Basic Linear Algebra Subprograms (BLAS; ) implemented in Automatically Tuned Linear Algebra Software (ATLAS; ) to perform basic vector and matrix operations. This has been found to increase the speed of PROCOV by at least three fold (see results below). Recommendations for utilizing the BLAS libraries other than through the free ATLAS (e.g., through the commercial Sun Performance Library or Intel^® ^Math Kernel Library) are included in the Makefile.

## Results

### Comparing the speedup of PROCOV with the new BLAS implementation

To compare the speedup of PROCOV with the BLAS implementation versus the non-BLAS implementation, we tested two protein datasets (Acetyl-CoA carboxylase with 36 taxa and 212 sites and Heat shock protein 70 (HSP70) with 34 taxa and 432 sites) for fixed topologies, previously inferred with PHYML [41] under JTT + Gamma, and optimized the parameters with JTT + the general covarion model with PROCOV. For Acetyl-CoA carboxylase, with the BLAS implementation, it took 22 minutes to finish parameter optimization and obtain the final log-likelihood score whereas the non-BLAS version took 1 hour 42 minutes for the same analysis. For the HSP70 data set with a fixed tree, the BLAS and non-BLAS versions took 49 minutes and 2 hours 28 minutes, respectively. The final likelihood scores yielded by the BLAS and non-BLAS versions of PROCOV are the same in both cases.

To assess the performance for PROCOV on tree searching, we simulated five datasets of 250 sites with seq-gen-aminocov [19] based on a tree topology obtained from a 17-taxon 60 KDa chaperonin (CPN60) dataset [10]. The reference tree and simulated datasets are available on the PROCOV web site. The simulations employed the JTT model and the RAS, Tuffley-Steel (TS), Galtier, Huelsenbeck and general covarion models, respectively. For the models with an RAS-process (all but the TS model), four gamma rates were used in simulation. We then used PROCOV to estimate the topology for each dataset under the corresponding model (i.e, using the same model that was used for simulating the data for each dataset) and (except for the TS model) 4 gamma rates, with a starting tree that was obtained with the neighbor-joining method by PHYLIP for each dataset. PROCOV successfully recovered the same true topology in each case. The speedups in PROCOV with the BLAS versus non-BLAS implementations are 1.6, 3.2, 3.4 and 3.8 fold for the four covarion models, respectively. There is no speedup for the RAS model, as the BLAS libraries are not implemented for calculations under the RAS model. The above comparative results with and without the use of the BLAS libraries were conducted on a computer with a 2.93 GHz Intel quad core Xeon processor with 15.69 GB RAM. Similar speedups were also observed on a computer with a different CPU architecture (2 GHz AMD Opteron processor with 2 GB RAM).

Table 1 shows for each simulated dataset the parameter estimates from tree search with the BLAS version of PROCOV. It appears that α was very well estimated in all models; π was also estimated well; covarion switching parameters were estimated fairly well in the simpler models (TS, Galtier and Huelsenbeck); they are less accurate in the general model, especially s11. Nevertheless, the rank order of the parameters used in the simulation, i.e., s01<s10<s11, is preserved in the estimates. This is consistent with larger variances previously observed in estimates of these parameters under the general covarion model [10]. Table 1 also lists the maximum likelihood of each dataset, the number of SPR trees searched and the CPU times used. The TS and RAS models spent the least time and the other three models spent about the same amount of time. The fact that the general model spent even less time than the Galtier model indicates that the running time is not only determined by the complexity (the number of free parameters) of the model but also how fast the program converges during each round of SPR search.

**Table 1:** Estimated parameters under SPR-based full tree search for datasets of 250 amino acid sites simulated based on a 17-taxa chaperonin tree with JTT + the listed models.

**Model**	**Parameter**			**ln likelihood**	**# SPR**	**CPU time**
				
		**Used in simulation**	**PROCOV estimation**		**trees searched**	
RAS	α	0.50	0.50	-4245.78	698	17 min

Tuffley-Steel	s01	1.875	1.66	-4752.23	648	14 min
				
	s10	1.25	0.80			

Galtier	α	0.5	0.41	-4188.25	673	6 hr 52 min
				
	s11	1.5	2.29			
				
	π	0.6	0.57			

Huelsenbeck	α	0.5	0.46	-4070.79	648	6 hr 8 min
				
	s01	1.875	1.89			
				
	s10	1.25	0.83			

General	α	0.5	0.50	-4156.24	672	6 hr 26 min
				
	s01	1.5	1.00			
				
	s10	2	1.35			
				
	s11	2.5	5.35			
				
	π	0.6	0.62			

**Figure 1:**
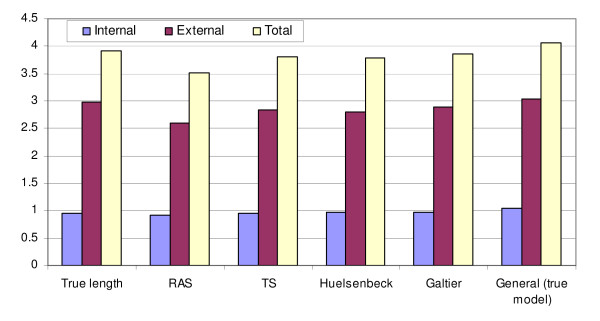
**The true tree length of a 17-taxa CPN60 tree used for simulating the datasets and the estimates under the different models**. The tree lengths (the Y axis) are shown as the sum of the external branches (External) and the sum of the internal branches (Internal) and the total tree length (Total) separately. The data was simulated under JTT + the general covarion model and estimated under the other models.

As the general covarion model takes much more time than the simpler covarion (e.g., TS) and RAS models in inferring a phylogeny, we asked whether the simpler models can correctly reconstruct phylogenies for data simulated under the general model. This is of practical importance as if it is true then the simpler models would prefer to be used to reconstruct the phylogenies to save computational time. For the CPN60 dataset simulated under the general model, we used each of the simpler models (RAS, TS, Galtier and Huelsenbeck) to estimate the phylogenies. Each of the models were able to correctly infer the same correct topology as using the general model but the running times were very different. The RAS and TS models took 16 and 4 minutes, respectively; both the Galtier and Huelsenbeck models took more than 5 hours. Despite the same topology inferred, branch length estimates were different from those that estimated under the general model, which were the true model that were used for simulation. Figure 1 shows the true tree length used for simulation and estimates under the RAS, TS, Galtier, Huelsenbeck and general models. The tree lengths were separated as the sum of the external branch lengths and the sum of the internal branch lengths. All simpler models, especially RAS, underestimated the branch lengths. Therefore, the general model may not be replaced by simpler models for certain types of data.

Previously we showed that the general model can converge to the Huelsenbeck and Galtier models when datasets are simulated under these models [10]. Here we further show that the general model can even adapt to the RAS model when the data are simulated under RAS. For the CPN60 dataset simulated under the JTT + RAS model, the general model recovered the same correct topology as the RAS model. Moreover, the branch length estimates under the general model are very close to that under the RAS model (the differences in the sums of the internal and external branch lengths are 0.01 and 0.03, respectively) for the total true tree length of 3.92. It turns out that the general model was able to adjust the covarion parameters (the covarion proportion π = 0.03, s01 = 0.03, s10 = 0 and s11 = 50, indicating no covarion for this data) to converge to the RAS model. Therefore, both the topology and the branch lengths were correctly inferred. For the same RAS-simulated dataset we also found that the Huelsenbeck model was able to correctly estimate both the topology and branch lengths by adjusting parameters to mimic RAS-like process (s01 = 100, s10 = 0).

### Establishing the phylogenetic position of Microsporidia

With the speedups of PROCOV made possible by the use of the BLAS libraries and other algorithmic improvements, it is now possible to compare topologies and, in some cases perform tree searches, for larger phylogenomic datasets (i.e. data sets made up of super-matrices of proteins) under covarion models in a reasonable time. Deep phylogenetic analyses of the eukaryote tree are often plagued with long-branch attraction (LBA) artifacts, even when large multi-gene phylogenomic data sets are used [42,43]. One of the most famous examples of this concerns the position of Microsporidia, a group of fast-evolving intracellular parasites that are now known to be relatives of Fungi. When reconstructing the phylogeny of eukaryotes rooted by Archaea, if the estimation is performed with ML under an RAS model, the extremely long branch leading to Microsporidia is usually attracted to the long branches leading to the Archaea at the base of the eukaryotes regardless of what amino acid substitution models are used. Many methods have been proposed to solve this problem, including selective taxon sampling, removal of fast-evolving proteins and saturated sites [42-44], accounting for covarion shifts [44], amino acid profile mixture modeling [45], branch length mixture modeling [17] and rare genomic changes of conserved amino acids-multiple substitutions [46]. Here we applied the general covarion model + WAG to a large eukaryote phylogenomic data set made up of 133 proteins from 40 taxa and 24294 sites [42] and calculated the log likelihoods of two competing trees: the LBA topology where Microsporidia groups with Archaea and, the correct topology where Microsporidia groups with Fungi. The general covarion model clearly supports the correct Microsporidia + Fungi tree with a large log-likelihood gain (4416.87) compared to the LBA tree (Table 2). In contrast, the correct tree has a smaller log-likelihood than the LBA tree under the RAS + WAG model (the log-likelihood difference between the right and the wrong trees is -197.5). Thus, for a real example, the covarion model appears to be less susceptible to the effects of LBA than the RAS model. We recently also used a site-specific class frequency mixture model implemented in QmmRAxML [47] to analyze the data and found the mixture model supported the correct topology, albeit with a smaller log likelihood gain (Table 2). Therefore, in this particular case, the LBA problem can be overcome with more realistic phylogenetic models that either account for site-specific substitution dynamics or covarion-like evolution.

**Table 2:** Log-likelihoods of the two competing trees of the Microsporidia data [42] calculated with PROCOV under the general covarion model (GCM) and the RAS model, respectively, and with QmmRAxML under the class frequency mixture model (cF) [47].

**Tree**	**PROCOV**		**QmmRAxML**
	
	**GCM+WAG**	**RAS+WAG**	**cF+RAS+WAG**
Microsporidia-fungi-clade	-737,304.13	-742,093.43	-731,758.97

Microsporidia-archaea-clan	-741,721.00	-741,895.93	-731,780.03

Log likelihood difference between the two trees	4416.87	-197.50	21.06

### Detecting covarion sites of functional and structural significance

Covarion models are useful not only because of improved phylogenetic estimation; they can also be used to identify patterns of sequence evolution that explain divergence in protein function or structure. Previous computational work on elongation factors (EF) has nicely demonstrated that identifying evolutionary site-rate shifts coupled with analyses of three-dimensional structures of the protein family can pinpoint sites that are likely important in functional divergence and structural change between bacterial elongation factor Tu (EF-Tu) and eukaryotic elongation factor 1α (EF-1α) [24]. In fact a number of additional methods have been developed over the last decade to identify rate-shifted sites for the same purpose [2,23,25-28,48]. Most of these methods rely upon assuming that a discrete shift in rates at many sites has occurred over one branch in the protein phylogeny under examination and estimation of the phylogeny is usually performed beforehand using standard phylogenetic models.

**Figure 2:**
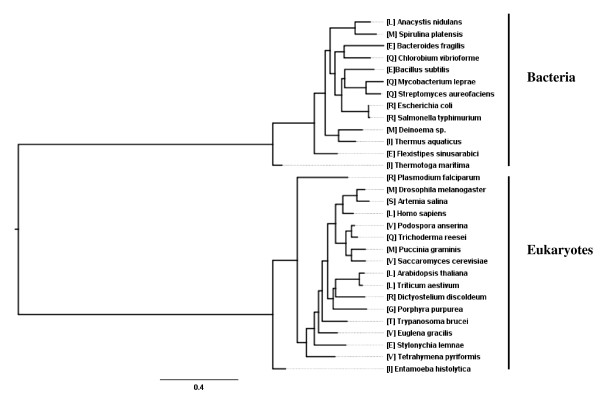
**A phylogenetic tree of the bacterial EF-Tu and eukaryotic EF-1α inferred with PROCOV under WAG + the general covarion model**. Brackets refer to the amino acids of the two groups at position 256, a site illustrating a non-typical covarion pattern where both eukaryotic (EF-1α) and bacterial (EF-Tu) sequences are very variable. Changes in EF-1α are more radical (i.e. substitutions between amino acid with different physicochemical properties) whereas those in EF-Tu are structurally more conserved changes.

Since PROCOV specifically models changing rates at sites during tree estimation, it may also be used to detect such rate shifts and has the added advantage that these rate shifts need not occur only on a single split in the phylogeny. To illustrate its utility, we reanalyzed the bacterial and eukaryotic EF data (30 taxa and 380 sites) described in Gaucher et al. [24]. We first inferred ML phylogenetic trees for this data using PROCOV under WAG + RAS and WAG + the general covarion model, respectively. We also used QmmRAxML [46] with the WAG + RAS model to obtain an ML tree. All the three methods estimated the same tree (Figure 2), (which is slightly different from the tree reported in [24]). The log-likelihood difference for this tree under the general covarion model versus the RAS model is 157.27 (p-value < 0.01 by a likelihood ratio test with 4 degrees of freedom), confirming that the EF data shows covarion-like properties [23,24]. The parameters estimated under the general covarion model are α = 1.1089; s01 = 0.4473; s10 = 0.2723; s11 = 0.2519 and π = 0.9652. As described in Wang et al. (2007) [10], under this model site likelihoods are computed separately for both the covarion model (l_cov) and the RAS model (l_ras) and then combined to get a total weighted likelihood of the site (π × l_cov + (1-B) × l_ras). In order to determine which sites show a strong 'covarion' pattern, the difference in log-likelihoods between the two models at a site can be calculated as Λ = ln(l_cov) - ln(l_ras); covarion sites are expected to have a Λ >> 0 as compared to sites that do not change rates across the tree.

**Figure 3:**
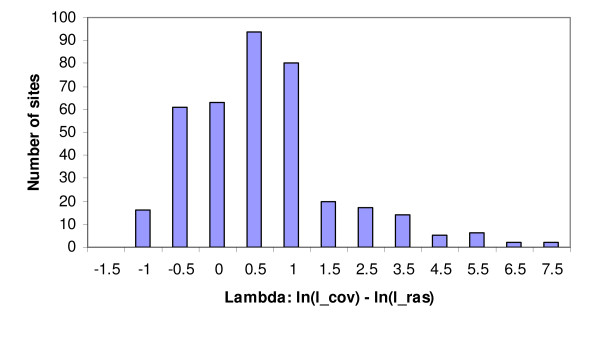
**The distribution of the difference between covarion log-likelihood and RAS log-likelihood at sites for the EF data analysed with the general covarion model**.

The -L option of PROCOV's command line arguments allows user to extract ln(l_cov) and ln(l_ras) for each site. We used this option to get the site likelihoods for the EF data under the general covarion model and calculated Λ for each site. Two hundred and forty out of the 380 sites have a positive Λ (i.e. ln(l_cov) > ln(l_ras)) while the remaining 140 sites have a negative Λ. Figure 3 shows a histogram of the distribution of Λ with a mean of 0.45 and standard deviation (SD) of 1.3. Twenty one sites have a Λ > mean + 2 SD and 42 sites have a Λ > mean + 1 SD. However, use of this distribution to identify covarion sites is not straightforward since the long right tail of the distribution (Figure 3) is likely due to the presence of many covarion sites. Thus the SD of this distribution is expected to be inflated relative to the SD of Λ distribution for non-covarion sites.

**Figure 4:**
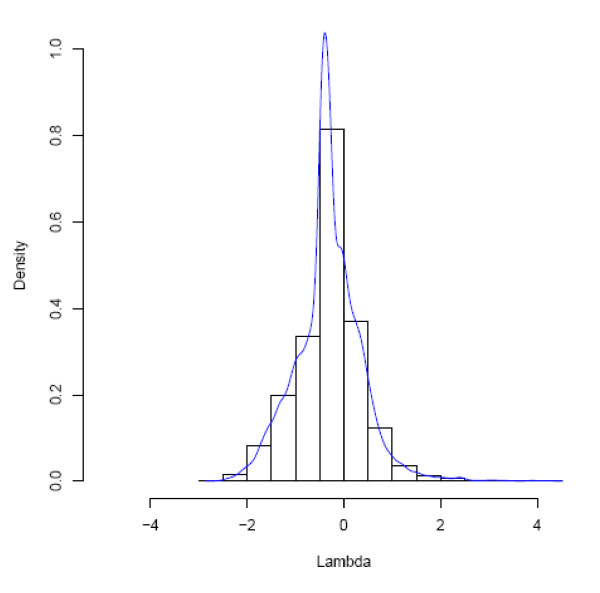
**A frequency density distribution of Λ, the difference between covarion log-likelihood and RAS log-likelihood at sites, estimated under the general covarion model for a dataset (10,000 sites) simulated under the RAS model based on the EF tree (shown in Figure 1)**.

In order to get a valid cutoff value for Λ that indicates a significant likelihood difference between the two models at the site (i.e., identify if the site is a covarion site), we used seq-gen-aminocov [19] to simulate 10,000 sites based on the EF tree (Figure 2) under the WAG + RAS model with 4 gamma rates and α = 0.8436, which is the fitted α for the original EF data estimated with the RAS model. We then used the general covarion model to calculate the covarion and RAS site likelihoods by fixing the topology and allowing all the parameters to be optimized. We calculated Λ for each of the 10,000 sites of the simulated EF data. Figure 4 shows the frequency distribution of Λ for the simulated data. The 99th percentile of the Λ distribution is 1.652, and can be used as a threshold for statistical significance. Note however this threshold value of 1.652 is model (tree and parameter) specific and therefore is only valid for the current original EF data. Using this criterion, 43 sites in the original EF dataset have a Λ greater than 1.652 which can be considered covarion sites with confidence at P < 0.01. As the dataset has 380 positions, one can expect about 4 (380 × 0.01) sites could be covarion sites (i.e. fall above the threshold value) by chance alone. This indicates there are many more sites with significant log likelihood differences than expected in the original EF dataset.

**Figure 5:**
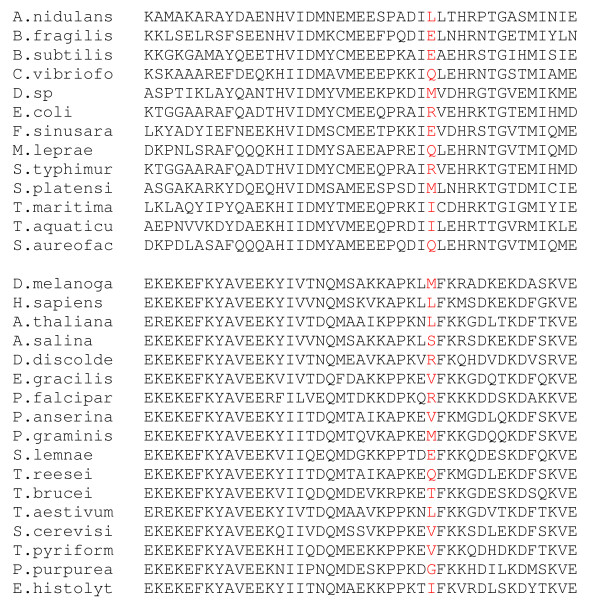
**Forty three sites that were detected by PROCOV as the covarion sites in the EF dataset (the upper part of the alignment is the bacterial EF-Tu and the lower part is the eukaryotic EF1α)**. The positions are 31, 32, 34 - 40, 42, 51, 64, 67, 69, 82, 96, 103, 106, 123, 131, 133, 138, 144, 153, 160, 163, 178, 189, 256, 263, 271, 288, 305, 311, 325 - 327, 329, 336, 350, 351 and 355, 356. Site 256 is shown in red.

The 43 covarion sites PROCOV identified constitute 11.3% of the total sites in the EF alignment. This estimate is consistent with some suggestions that about 10% of the sites in this data set are covarion sites [24]. However, the EF dataset used here is a relatively small one with 17 EF-1α and 13 EF-Tu sequences. Inclusion of more taxa would be expected to increase the proportion of covarion sites detected [23,27,49]. In any case, Table 3 shows a list of the 43 identified positions in descending order of Λ. The sequence alignment columns corresponding to these sites are shown in Figure 5. Twenty four of the sites (marked as 'c' in the 'Covarion site' column in Table 3) are the sites that were previously identified to be covarion sites of functional/structural significance by Gaucher et al. [24]. Each of these displays a typical 'covarion-like' site pattern, i.e., a lot of different amino acid states occur in the bacterial group but no or little change occurs at that site in the eukaryotic group or *vice versa*. Eighteen sites (marked as 'c1') were detected as covarion sites by PROCOV also demonstrate this typical covarion pattern but were not flagged by Gaucher and colleagues' method. As an independent test, we also used our rate-shift detection program Bivar [13] to estimate rate differences between the two subgroups of EF-Tu and EF-1α, which recovered 34 sites as rate shifted with a p-value < 0.05. Thirty one of these are the same covarion sites as picked up by PROCOV (Table 3). Eleven sites (32, 37, 39, 67, 96, 106, 160, 178, 271, 350 and 356) identified by PROCOV as covarion sites clearly show a typical covarion pattern, but these sites were not picked up by Bivar (p-value > 0.05 in Table 3). These comparisons indicate PROCOV may have more power to identify covarion sites than either Bivar or the Gaucher et al. method.

**Table 3:** Forty three sequence positions in the EF data show the highest differences between covarion site likelihood and RAS site likelihood.

	Position*	Lambda**	Covarion site***	Bivar P-value****
1	34	7.280	c	<0.001
2	36	6.503	c	<0.001
3	325	6.246	c	<0.001
4	305	5.652	c	<0.001
5	138	5.458	c	<0.001
6	336	4.873	c	<0.001
7	329	4.790	c	<0.001
8	153	4.702	c	<0.001
9	327	4.632	c	0.014
10	35	4.595	c	0.022
11	123	4.438	c	0.004
12	311	4.199	c	0.003
13	189	4.034	c	0.007
14	103	3.906	c	0.001
15	69	3.726	c	0.004
16	131	3.430	c	0.002
17	256	3.256	c2	0.122
18	351	3.202	c	0.027
19	38	3.120	c1	0.043
20	51	3.073	c1	0.013
21	42	3.057	c1	0.029
22	106	2.896	c1	0.064
23	67	2.866	c	0.120
24	271	2.793	c1	0.064
25	133	2.789	c	0.045
26	144	2.700	c1	0.002
27	163	2.604	c	0.006
28	263	2.588	c	0.033
29	31	2.557	c1	0.012
30	39	2.442	c1	0.065
31	160	2.274	c	0.073
32	64	2.23	c1	0.038
33	32	2.143	c	0.147
34	82	2.116	c1	0.029
35	96	2.092	c1	0.080
36	326	2.060	c	0.021
37	178	2.047	c1	0.341
38	37	1.935	c1	0.081
39	40	1.921	c1	0.023
40	350	1.875	c1	0.071
41	355	1.818	c1	0.039
42	288	1.755656	c1	0.043
43	356	1.694422	c1	0.111

* Sequence position is based on the EF alignment [24]. **Λ = ln(l_cov_) - ln(l_ras_). ***c: sites were found to be covarion sites of functional or structural significance in [24]; c1: sites having typical covarion site pattern but missed in [24]; c2: site showing non-typical covarion site pattern. ****P-values from a Bivar analysis of the EF data.

Site 256 in Table 3 is particularly interesting, as it ranks relatively high (17^th^) among the log-likelihood differences between a covarion process and the RAS process yet has a non-significant Bivar p-value of 0.12. The method used by Gaucher and colleagues also did not pick up this site as a covarion site. Inspection of the residues at this site (Figure 5), reveals that it does not have a typical 'covarion pattern' as the site is variable in both bacterial and eukaryotic EFs. The EF-1α subgroup is slightly more variable at this site, displaying 10 different amino acids that collectively can be binned into 4 of the six different "Dayhoff" groups of amino acids (I, L, M, V; E, Q; R; G, S, T) as compared to the EF-Tu subgroup, which has 6 different amino acids from 3 of the Dayhoff groups (I, L, M; E, Q; R) [50,51]. Figure 2 shows the amino acids at site 256 mapped on to the EF-Tu/1α tree. Close inspection of the substitutions at this site in the EF-1α subtree reveals that a number of radical amino acid changes occur between relatively closely related sister taxa in the tree (e.g. *Drosophila *has an M, versus S in *Artemia *and *Podospora *has a V where *Trichoderma *has a Q). Such radical changes are not observed in similarly closely related bacteria in the EF-Tu subtree. A subsequent analysis of the two subgroups separately with the general covarion model indicates the eukaryotic EF1α has a very big positive difference between ln(l_cov) and ln(l_ras) at site 256 (Λ_EF-1α _= 6.35) which suggests it could be a covarion site for the eukaryotic subset. By contrast, Λ_EF-Tu _= 1.55 for the bacterial subset and is unlikely a covarion site, although a simulation study is needed to determine a Λ threshold for the two subtrees separately.

Despite the strong support for site 256 being a covarion site in EF-1α, the residues at the site do not present a typical covarion pattern where variability is differentially restricted in different groups. One possible explanation of these observations is that the covarion model is compensating for the radical substitutions between closely related taxa observed in the EF-1α subtree, which are not consistent with the WAG substitution model. A rate-switching process could accommodate such radical substitutions by in effect 'lengthening' the branches between closely related taxa. This is in contrast to an RAS model where the rates of evolution must remain constant across the tree even if radical substitutions are observed in some closely-related taxa but not in others. To test the idea that the covarion model was compensating for this kind of substitution model misspecification at site 256, we compared the likelihood of this site under a simple proportional (Prop + RAS) model (where substitution rates are proportional only to the target amino acid frequencies in the data set) relative to the site likelihood under the WAG + RAS model. As expected, for EF-1α site 256, the ln(l_prop+ras) = -54.83 which is greater than ln(l_wag+ras) = -56.34, despite the fact that over all sites the WAG + RAS model has a greater log-likelihood (-12.51 per site) than the Prop + RAS model (-13.63 per site) for this subgroup. This result suggests that it is the low exchangeability rates in the WAG model corresponding to the radical amino acid changes observed at this site that lead to the poor model fit. Although unexpected, it seems that the covarion model compensates for this kind of model misspecification at sites that do not show classical covarion-type variability patterns.

**Figure 6:**
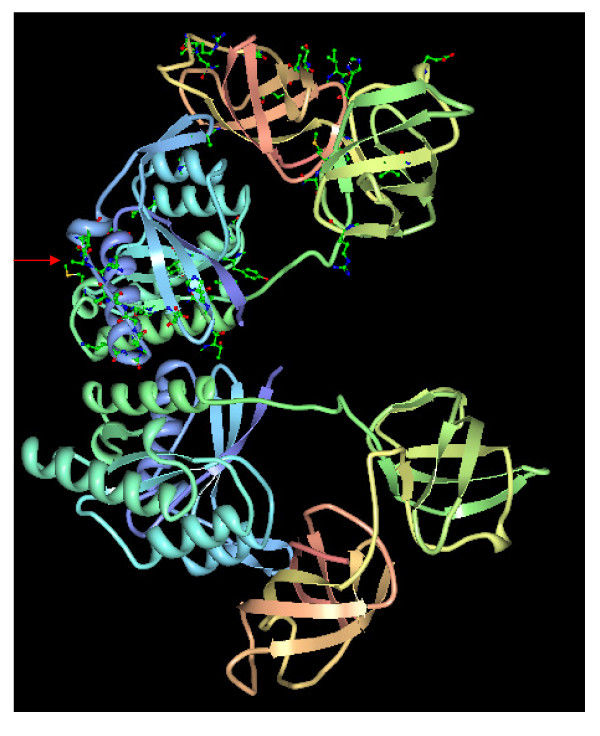
**Tertiary structure of E. coli EF-Tu (PDB ID: **1EFC) [57], **which has two identical polymer chains (A and B)**. The covarion residues are mapped on the A chain (the top polymer). The red arrow points to the purple strip of a loop region of 10 nearly consecutive covarion sites (sites 37, 38, 40 - 46, 48 in 1EFC), which corresponds to sites 31, 32, 34 - 40, 42 on the EF alignment listed in Figure 5. The loop region is connected to the two helices, one at either end.

In any case, although the 43 covarion sites listed in Table 3 are scattered throughout the 380 sequence positions in the alignment, they are not randomly distributed. For instance, there is a long sequence segment (sites 31 - 42) that, with the exception of sites 33 and 41, are all covarions. This segment maps to a surface loop region of the EF-Tu structure (Figure 6), that is a possible ribosome binding site in bacteria [24]. The pattern at these sites indicates that the bacterial EF-Tu sequences are typically variable whereas the eukaryotic EF-1α sequences are conserved, hinting a possible additional function for this loop in eukaryotes that is absent in bacteria.

## Discussion

We have developed PROCOV, an ML-based phylogenetic program for modeling the covarion processes of protein evolution. We showed that compiler optimization, especially the use of highly optimized math libraries, such as BLAS, can significantly speed up likelihood calculation. Although BLAS and related math libraries have been widely used in high performance computing software (e.g. Matlab and R), we are not aware of other phylogenetic software that utilize these efficient libraries. The use of the optimized math library together with some features of PROCOV described above makes it tractable to do full tree search under the general covarion model for datasets of moderate size in a reasonable time (Table 1). For large datasets one can selectively restrict the movements of those branches and nodes that deem to be in the same group when running PROCOV. This partial search will considerably reduce tree search time when many nodes and branches are fixed. For even larger phylogenomic data one can use PROCOV to analyse several competing trees that were already established by other phylogenetic methods and see which of them is preferred by the general model. We applied this method to the Microsporidia phylogenomic dataset [42] and the general model clearly supports the correct Microsporidia-fungi clade tree over the LBA-induced Microsporidia-anchaea clan tree. However, this may not guarantee it is the optimal tree for the general model if a tree search is conducted. For example, a partial tree search of this Microsporidia data estimated a tree of Microsporidia-protist clade that had a higher likelihood than the tree of Microsporidia-fungi clade.

Examples in this study show that phylogenetic tree estimation under a covarion model may or may not estimate a different optimal topology than that under a non-covarion RAS model. For the simulated CPN60 datasets as well as the EF dataset, the RAS and covarion models estimated the same optimal topologies; for the Microsporidia data they differ. Our previous simulations and analytical studies explored topology and branch length conditions that the RAS and covarion models will likely estimate different topologies [19]. Results in Figure 1 show that even though the RAS model was able to estimate the correct topology for data simulated under the general model, it would underestimate the branch lengths. Both the general and Huelsenbeck models, however, will correctly infer the topology and accurately estimate branch lengths for the data simulated under the RAS model. They do so by adjusting the covarion parameters to converge to the RAS model. For real data, we do not know in advance whether the data follow covarion or RAS evolutions or both. The general model, including the RAS and TS, Huelsenbeck and Galtier models as special cases, has the advantage of adapting to the right model in the course of parameter optimization so that it can analyse all relevant types of data appropriately, but suffers from heavy computing loads with large amounts of data.

A recent empirical test of the covarion hypothesis has shown that the frequency of covarion-sites increases with genetic distance [52]. This suggests covarion-based phylogenetic inference may be useful in the estimation of the divergence time of the species spanning longer time periods. It will therefore be interesting to revisit the estimates of dates of divergence using relaxed molecular clock methods [53] in conjunction with covarion models of evolution.

In addition to the advantages of PROCOV for phylogenetic inference under the general model, we also demonstrated that it had more power to detect covarion sites than several previous methods. It can also be used to pinpoint those lineages where covarions are located (data not shown). Like the general covarion model, covarion and RAS site likelihoods are also separately calculated under the Galtier model. By contrast, the TS models is not a mixture of covarion and RAS processes; the Huelsenbeck model, as originally formulated, does not calculate covarion and RAS site likelihoods, separately. Therefore only covarion site likelihoods are calculated for the TS and Huelsenbeck models. Nevertheless, one can run two separate analyses with PROCOV, one under either of the two models, another under the RAS model, and compare their site likelihood differences to obtain Λ's for sites.

All of the four covarion models considered here are stationary time reversible models with an expectation that the proportion of variable sites (p_var_) is the same in all evolutionary lineages. However, this assumption can be overly restrictive as proportions of variable sites may vary in different lineages [22,54]. A sequence generator for generating lineage-specific variation in the p_var _is recently reported [55]. A fruitful area of future development of PROCOV may therefore be to model both changes in the proportion of variable sites and the covarion-based rate changes and switches. Furthermore, the current implemented covarion models assume that rate switching between 'on' and 'off' states and among different 'on' rates are homogenous across sites and the tree, which may not be realistic. This is especially suspicious for large phylogenomic datasets that are from the concatenation of multiple genes of diverse functions with different functional constraints. For instance, we previously reported that the covarion parameters, like the α parameter of the RAS process, varied across different protein families (see Supplementary Table one of [10]). It will be interesting to model this heterogeneity in switch rates variation across sites and lineages and implement it in PROCOV without increasing computational load too much. Finally, the current release of PROCOV (version 2.0) only handles protein sequence data. Analyses of DNA substitutions under covarion models have found applications in inferring the evolutionary history of viral genes [30,31,56]. Future extension of PROCOV to allow analyses of DNA sequence data may be useful to investigate these kinds of data sets.

## Conclusion

PROCOV is a phylogenetic program to infer phylogeny under covarion models, which may be especially useful for problems involving estimates of deep divergences in the tree of life, where rates of evolution at sites are likely to have changed over the tree. It can also be used to detect covarion sites, which when combined with structural bioinformatics approaches, can be a powerful method to study lineage-specific functional shifts in protein families as well as protein adaptation.

## Availability and requirements

* Project name: PROCOV: maximum likelihood estimation of protein phylogeny under covarion models (version 2.0).

* Project home page: .

* Operating system(s): Any Unix/Linux platform.

* Programming language: C.

* Other requirements: GCC (version 3 or higher) or compatible compiler. It is recommended to have the BLAS/ATLAS libraries  installed on the Unix/Linux system so that PROCOV can run faster. Versions of BLAS and LAPACK, such as the generic versions from ATLAS, Netlib, or vendor-provided libraries that work with your compiler should be installed. The Makefile should then be edited to match the type of the compiler and the path and library names of the BLAS and LAPACK libraries used. The Makefile of the PROCOV source code gives some instances of the BLAS installation on a few commonly-used unix systems.

* License: GNU GPL.

* Any restrictions to use by non-academics: None.

## Authors' contributions

All authors conceived of the study, designed the method and wrote the article. H-CW implemented the software and ran the analyses.
